# Massive Desmoplastic Fibroma of the Proximal Tibia: Case Report

**DOI:** 10.1055/s-0043-1771492

**Published:** 2024-02-01

**Authors:** Oriol Pujol, Sara Castellanos, María G. Carrasco, Alejandro Garzón, Cleofe Romagosa, Roberto Vélez

**Affiliations:** 1 Unidade de Oncologia Ortopédica, Departamento de Cirurgia Ortopédica, Hospital Universitari Vall d'Hebron, Barcelona, Spain. Universitat Autónoma de Barcelona. Barcelona, Spain; 2 Departamento Anatomopatológico, Hospital Universitari Vall d'Hebron, Barcelona. Universitat Autónoma de Barcelona. Barcelona, Spain

**Keywords:** bone neoplasms, case report, fibroma, desmoplastic, orthopedic, tibia

## Abstract

Desmoplastic fibroma of bone is a very uncommon, benign but locally aggressive fibrogenic tumor. This report describes the case of a 45-year-old patient with a massive desmoplastic fibroma of the proximal tibia. A two-staged surgical procedure was successfully performed: wide resection and endoprosthetic reconstruction. Surgeons should be aware of the complexity of its treatment in the locally advanced and aggressive cases. A comprehensive review of the literature is also provided.

## Introduction

Desmoplastic fibroma of bone is a benign but locally aggressive fibrogenic tumor. It is one of the less common primary bone tumor (<0.1%). 1 It tends to occur in adolescent and young adult patients, with equal sex distribution 2 This lesion shows a predilection for craniofacial bones; however, long bones are also commonly involved, especially the distal femur and proximal tibia. 3

There is a scarcity of literature on the features and management of this very rare bone tumor. We report the case of a 45-year-old patient with a massive desmoplastic fibroma of the proximal tibia.

## Case Report

This case report was approved by our institution (PR(AT)95/2022). Informed consent was obtained from the patient.

A 45-year-old male patient was referred to our clinic for evaluation of a symptomatic lytic lesion in the proximal right tibia. Previously, he had been treated and followed-up in another center. There, a benign bone tumor had been diagnosed 27 years before. Curettage had been performed twice, but the tumor recurred. During previous annual follow-ups, he had been painless and had presented almost complete functionality. Since his life quality had not been affected and the lesion had been considered a benign tumor, the patient had not consented to surgery.

**Fig. 1:**
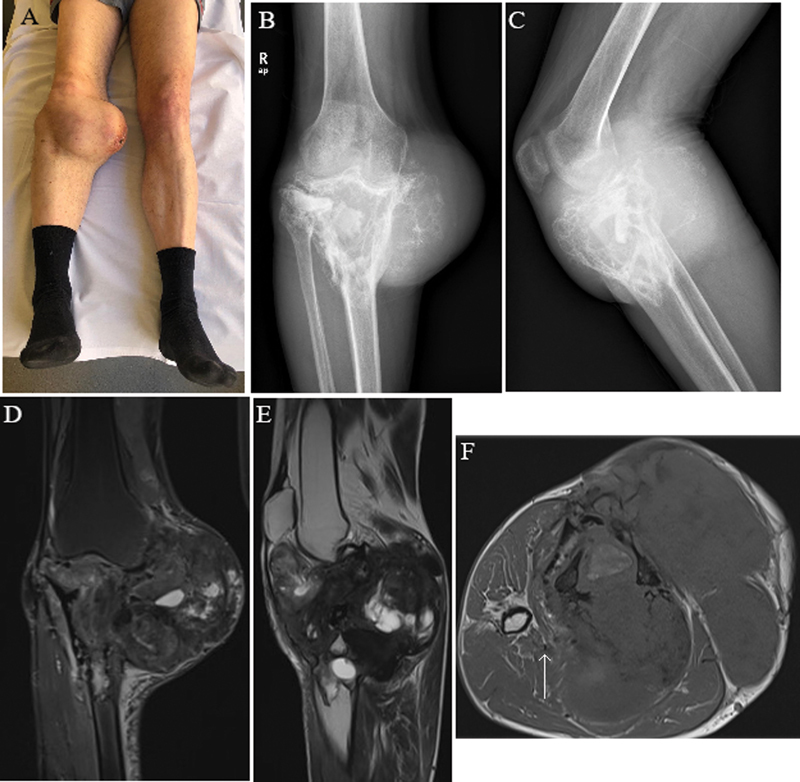
Preoperative images of the patient. A) The clinical photograph shows an evident knee deformity, with a hard palpable medial mass measuring 10cm. B and C) Radiographic evaluation shows a massive lytic tumor in the proximal tibia. The lesion presented an aggressive growth with bone destruction, trabeculation and extension into the fibula and the surrounding soft tissue. D, E and F) MRI images demonstrated a 13 × 15 × 13 cm mass (AP × ML × CC) with significant soft tissue involvement and displacement of neurovascular structures (white arrow). Heterogenous contrast enhancement can be observed.

On presentation at our center, physical examination showed evident knee deformity, with a hard palpable medial mass measuring 10cm ( Fig. 1a ). The patient referred pain with activity and loss of functionality. X-ray images showed a massive lytic tumor in the proximal tibia causing bone destruction ( Fig. 1b-c ). MRI images demonstrated a 13 × 15 × 13cm mass (APxMLxCC) with significant soft tissue involvement and displacement of neurovascular structures ( Fig. 1d-f ). An ultrasound-guided biopsy for histological analysis was performed; the definitive diagnosis was desmoplastic fibroma of bone. After discussing the case in the multidisciplinary committee, a two-staged surgical procedure was deemed the best option.

**Fig. 2:**
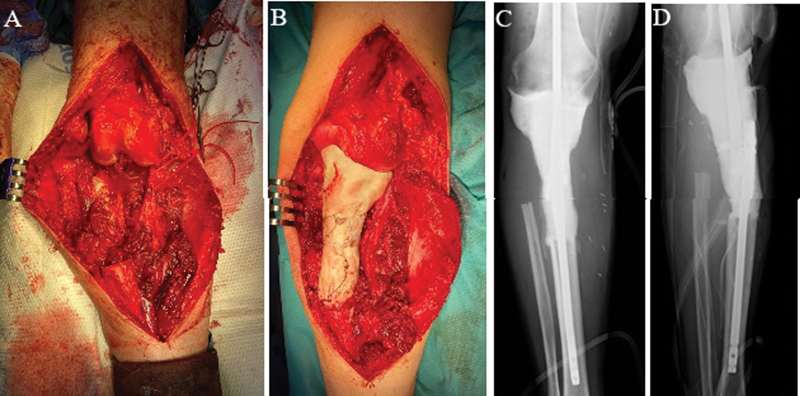
Images of the first surgical stage. A) The intraoperative photograph shows the tumour wide margin resection. The neurovascular bundle was identified, dissected and protected. A relevant dead space can be observed. B, C and D) The intraoperative photograph and the postoperative x-ray images show the temporary arthrodesis performed with two intramedullary nails (anterograde tibial and retrograde femoral). Antibiotic loaded cement was used to increase the construct stability, obliterate the dead space and locally deliver antibiotic.

**Fig. 3:**
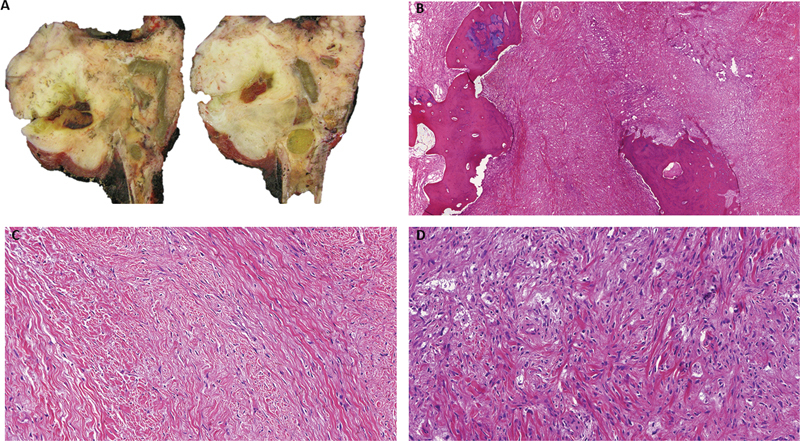
Images of the anatomopathological study of the surgical specimen. Negative margins were demonstrated. No necrosis was observed and only two mitoses per 10-HPF were counted. Immunohistochemically, the tumor cells were positive for MDM2 and negative for sm-actin, desmin, beta-catenin, S100, CD34. FISH for MDM2 showed a polysomy, but not amplification. A) Macroscopic image of the sectioned surgical specimen showing the tibia with a white lobulated tumor that extends to soft tissues with hemorrhagic areas. B) Infiltration of the lamellar bone by the neoplasm. Image at 50x of H&E. C) Hypocellular proliferation of spindle cells with bland nuclear features organized in a longitudinal fascicular pattern on a collagenized stroma. Image at 400x of H&E. D) Few areas showing mildly atypical cells arranged in a disordered pattern. Image at 400x of H&E.

The first stage consisted on resecting the tumor with wide surgical margins. In supine position and under tourniquet, an anterior approach with an extended medial parapatellar arthrotomy was performed. Only the anterior tibial artery was ligated; while the other main branches of the popliteal artery and the sciatic nerve were identified and preserved ( Fig. 2a ). A wide excision of the tumor was performed. Femur was preserved during this surgical stage since it was not affected by the tumor. Tibia was resected with 3cm wide margin. Then, a temporary arthrodesis was performed ( Fig. 2b-d ). Local flap coverage using the medial gastrocnemius was required. The anatomopathological study of the surgical specimen ( Fig. 3a-d ) confirmed the diagnosis of desmoplastic fibroma of bone and demonstrated negative margins. Postoperatively, the patient presented a foot drop caused by a common peroneal nerve palsy. It was treated with physical therapy and antiequine orthosis.

The second stage was performed seven months later, aiming to achieve a functional limb reconstruction using an endoprosthetic replacement. In supine position and under tourniquet, the same anterior approach was performed. First, the cement spacer and the nails were removed. Then, the prosthesis was implanted according to the usual technique. However, on deflation of the tourniquet, a pulsatile bleeding from the posterior aspect of the knee was seen. Tourniquet was inflated again and a complete laceration of the popliteal artery was identified. An arterial bypass using a greater saphenous vein autologous graft was successfully accomplished by a vascular surgeon.

**Fig. 4:**
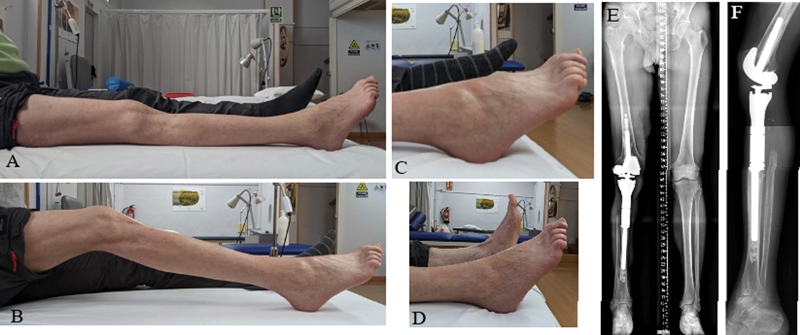
Final postoperative images. A) Knee extension , B) Knee flexion C) Full plantar flexion D) Dorsal flexion deficit (−40°). E and F) Postoperative full-length and lateral x-ray images show a correct implantation of the endoprosthetic replacement.

After 18 months of follow-up, the tumor has not relapsed and no complications have occurred. The patient is pain-free and has resumed his normal daily activities. However, he presents a restricted knee ROM (0-30°) and a foot drop (40° dorsal flexion deficit) ( Fig. 4 ). The patient is satisfied with the procedure (SAPS scale 22/28).

## Discussion

The patient presented a desmoplastic fibroma of the proximal tibia that has been followed up annually during the last 25 years. Despite the impressive radiological images, the patient remained asymptomatic until the last months. So far, surgical options were left aside because the patient did not consent to surgery. Long follow-up with conservative treatment has been previously reported in a locally advanced desmoplastic fibroma of the ilium. 4 Although desmoplastic fibroma is frequently asymptomatic (incidental finding), it may cause nonspecific symptoms such as swelling, pain, deformity or loss of function. 2

Patient's radiological images were compatible with the most aggressive pattern of desmoplastic fibroma; showing bone destruction, soft tissue extension and neurovascular structures displacement. On radiographs, desmoplastic fibroma usually presents as a lobulated and trabeculated well-defined lytic lesion with a narrow transition zone. It may also have an expansile growth, even breaching the cortex and extending into the surrounding soft tissue. The disease extension and margins are best assessed with MRI, being the ideal technique to describe soft tissue involvement. Desmoplastic fibroma typically shows a low to intermediate signal intensity foci on T2, which radiographically does not correspond to calcification. 5 Furthermore, it presents heterogenous enhancement after contrast administration.

Anatomopathological study provided the definitive diagnosis of our patient's tumor. The WHO described the desmoplastic fibroma microscopic appearance as being composed of spindle cells in a richly collagenous matrix. 1 The main differential diagnosis is a well-differentiated low-grade fibrosarcoma. However, desmoplastic fibroma presents minimal cytological atypia, pleomorphism and mitoses. Macroscopically, the tumor is firm and presents a creamy-white surface with a variegated whorled pattern.

Recurrence following simple curettage has been reported to very high; in consequence, surgical wide excision is the current treatment of choice. 6 7 8 9 Evans et al. 10 reported a recurrence rate after intralesional resection of 33%, while more aggressive techniques showed a 0% rate. They also stated that local recurrence was strongly associated with soft tissue extension. Böhm et al. 11 analyzed 189 patients and found a recurrence rate of 55-72% after simple curettage and 0% after wide resection. Furthermore, they highlighted the aggressive character that can present this entity due to its risk of pathological fracture (12%) and high recurrence rate.

On the other hand, good results after intralesional resection with less postoperative morbidity have also been defended. 12 13 Some authors stated that adding adjuvant radiotherapy to intralesional curettage may be an alternative approach when wide resection would lead to significant functional impairment. 14 During our aggressive surgical approach, the patient suffered a common peroneal nerve palsy and a popliteal vascular injury. Wide resection in massive tumors is not without complications. However, we preferred this approach because the tumor size, severe bone destruction, soft tissue extension and articular involvement made extremely difficult to perform a successful intralesional curettage without functional impairment or local recurrence.

We reported the case of a 45-year-old patient with a massive desmoplastic fibroma of the proximal tibia. A two-staged surgical procedure was successfully performed: wide resection and endoprosthetic reconstruction. Although desmoplastic fibroma is a benign tumor, surgeons should be aware of the complexity of its treatment in the locally advanced and aggressive cases.
