# Supplementary material for: Massive Desmoplastic Fibroma of the Proximal Tibia: Case Report
Source: Rev Bras Ortop (Sao Paulo). 2024 Feb 1;59(Suppl 1):e114–8. doi: 10.1055/s-0043-1771492 (PMC11254431; doi:10.1055/s-0043-1771492)
Supplement: Related texts

# Research article: Fibroma desmoplásico na tíbia proximal: Relato de caso

**Keywords:** fibroma desmoplásico, neoplasias ósseas, ortopedia, relato de caso, tíbia

## Abstract

O fibroma desmoplásico do osso é um tumor fibrogênico muito incomum, benigno, mas localmente agressivo. Este relato descreve o caso de uma paciente de 45 anos com um enorme fibroma desmoplásico na tíbia proximal. Foi realizado com sucesso um procedimento cirúrgico em dois tempos: ressecção ampla e reconstrução endoprotética. Os cirurgiões devem estar cientes da complexidade de seu tratamento nos casos localmente avançados e agressivos. Uma revisão abrangente da literatura também é fornecida.

## Introdução

O fibroma desmoplásico do osso é um tumor fibrogênico benigno, mas localmente agressivo. É um dos tumores ósseos primários menos comuns (<0.1%). 1 Tende a ocorrer em pacientes adolescentes e adultos jovens, com igual distribuição por sexo 2 . Essa lesão apresenta predileção pelos ossos craniofaciais; no entanto, os ossos longos também são comumente envolvidos, especialmente o fêmur distal e a tíbia proximal. 3

Existe uma escassez de literatura sobre as características e manejo deste tumor ósseo muito raro. Relatamos o caso de uma paciente de 45 anos com fibroma desmoplásico massivo da tíbia proximal.

## Relato de Caso

Este relato de caso foi aprovado por nossa instituição (PR(AT)95/2022). O consentimento informado do paciente foi obtido.

Paciente do sexo masculino, 45 anos, encaminhado ao nosso ambulatório para avaliação de lesão lítica sintomática na tíbia direita proximal. Anteriormente, ele havia sido tratado e acompanhado em outro centro. Lá, um tumor ósseo benigno havia sido diagnosticado 27 anos antes. A curetagem havia sido realizada duas vezes, mas o tumor voltou. Nos acompanhamentos anuais anteriores, apresentava-se indolor e com funcionalidade quase total. Como sua qualidade de vida não havia sido afetada e a lesão era considerada um tumor benigno, o paciente não havia consentido com a cirurgia.

Na apresentação em nosso centro, o exame físico mostrou deformidade evidente no joelho, com uma massa dura palpável medindo 10cm ( Fig. 1a ). O paciente apresentava dor com atividade e perda da funcionalidade. As radiografias mostraram um tumor lítico enorme na tíbia proximal causando destruição óssea ( Fig. 1b-c ). As imagens de ressonância magnética demonstraram uma massa de 13 × 15 × 13cm (APxMLxCC) com envolvimento significativo de tecidos moles e deslocamento de estruturas neurovasculares ( Fig. 1d-f ). Foi realizada biópsia guiada por ultrassom para análise histológica; o diagnóstico definitivo foi de fibroma desmoplásico do osso. Após discussão do caso em comissão multidisciplinar, o procedimento cirúrgico em dois tempos foi considerado a melhor opção.

**Fig. 1:** Imagens pré-operatórias do paciente. A) A fotografia clínica mostra uma evidente deformidade no joelho, com uma massa dura mediana palpável medindo 10cm. B e C) A avaliação radiográfica mostra um tumor lítico enorme na tíbia proximal. A lesão apresentava crescimento agressivo com destruição óssea, trabeculação e extensão para a fíbula e tecidos moles circundantes. D, E e F) Imagens de RM demonstraram uma massa de 13 × 15 × 13 cm (AP x ML x CC) com envolvimento significativo de partes moles e deslocamento de estruturas neurovasculares (seta branca). O realce heterogêneo do contraste pode ser observado.

A primeira etapa consistiu na ressecção do tumor de amplas margens cirúrgicas. Em decúbito dorsal e com torniquete, foi realizada abordagem anterior com artrotomia parapatelar medial estendida. Apenas a artéria tibial anterior foi ligada; enquanto os outros ramos principais da artéria poplítea e do nervo ciático foram identificados e preservados ( Fig. 2a ). Foi realizada excisão ampla do tumor. O fêmur foi preservado nesta etapa cirúrgica por não ter sido acometido pelo tumor. A tíbia foi ressecada com margem de 3cm de largura. Em seguida, foi realizada uma artrodese temporária ( Fig. 2b-d ). Foi necessário o uso de retalho do músculo gastrocnêmio medial para cobertura. O estudo anatomopatológico da peça cirúrgica ( Fig. 3a-d ) confirmou o diagnóstico de fibroma desmoplásico do osso e demonstrou margens negativas. No pós-operatório, o paciente apresentou pé caído causado por paralisia do nervo fibular comum. Foi tratado com fisioterapia e órtese antiequina.

**Fig. 2:** Imagens da primeira etapa cirúrgica. A) A fotografia intraoperatória mostra a ressecção com margem ampla do tumor. O feixe neurovascular foi identificado, dissecado e protegido. Um espaço morto relevante pode ser observado. B, C e D) A fotografia intraoperatória e as radiografias pós-operatórias mostram a artrodese temporária realizada com duas hastes intramedulares (tibial anterógrada e femoral retrógrada). Cimento carregado com antibiótico foi usado para aumentar a estabilidade da construção, obliterar o espaço morto e fornecer antibiótico localmente.

**Fig. 3:** Imagens do estudo anatomopatológico da peça cirúrgica. Margens negativas foram demonstradas. Nenhuma necrose foi observada e apenas duas mitoses por 10-HPF foram contadas. Imuno-histoquimicamente, as células tumorais foram positivas para MDM2 e negativas para sm-actina, desmina, beta-catenina, S100, CD34. FISH para MDM2 mostrou uma polissomia, mas não amplificação. A) Imagem macroscópica da peça cirúrgica seccionada mostrando a tíbia com tumor lobulado branco que se estende a partes moles com áreas hemorrágicas. B) Infiltração do osso lamelar pela neoplasia. Imagem em 50x de H&E. C) Proliferação hipocelular de células fusiformes com características nucleares suaves organizadas em um padrão fascicular longitudinal em um estroma colagenizado. Imagem em 400x de H&E. D) Poucas áreas mostrando células levemente atípicas dispostas em padrão desordenado. Imagem em 400x de H&E.

A segunda etapa foi realizada sete meses depois, com o objetivo de obter uma reconstrução funcional do membro usando uma substituição endoprotética. Em decúbito dorsal e sob torniquete, foi realizada a mesma abordagem anterior. Primeiramente, o espaçador de cimento e os pregos foram removidos. Em seguida, a prótese foi implantada conforme técnica usual. No entanto, ao esvaziar o torniquete, observou-se sangramento pulsátil na face posterior do joelho. O torniquete foi novamente insuflado e identificada laceração completa da artéria poplítea. Um bypass arterial com enxerto autólogo de veia safena magna foi realizado com sucesso por um cirurgião vascular.

Após 18 meses de acompanhamento, o tumor não recidivou e não ocorreram complicações. O paciente está sem dor e retomou suas atividades diárias normais. Porém, apresenta restrição da ADM do joelho (0-30°) e pé caído (déficit de flexão dorsal de 40°) ( Fig. 4 ). O paciente está satisfeito com o procedimento (escala SAPS 22/28).

**Fig. 4:** Imagens pós-operatórias finais. A) Extensão de joelho, B) Flexão de joelho C) Flexão plantar completa D) Déficit de flexão dorsal (−40°). E e F) Radiografias pós-operatórias de comprimento total e lateral mostram uma implantação correta da substituição da endoprótese.

## Discussão

O paciente apresentava um fibroma desmoplásico na tíbia proximal que vinha sendo acompanhado anualmente nos últimos 25 anos. Apesar das imagens radiológicas impressionantes, o paciente manteve-se assintomático até os últimos meses. Até agora, as opções cirúrgicas foram deixadas de lado porque o paciente não consentiu com a cirurgia. Longo seguimento com tratamento conservador foi previamente relatado em um fibroma desmoplásico localmente avançado do ílio. 4 Embora o fibroma desmoplásico seja frequentemente assintomático (achado incidental), ele pode causar sintomas inespecíficos, como inchaço, dor, deformidade ou perda de função. 2

As imagens radiológicas do paciente eram compatíveis com o padrão mais agressivo de fibroma desmoplásico, mostrando destruição óssea, extensão de tecidos moles e deslocamento de estruturas neurovasculares. Nas radiografias, o fibroma desmoplásico geralmente se apresenta como uma lesão lítica bem definida, lobulada e trabeculada, com uma zona de transição estreita. Também pode ter um crescimento expansivo, até rompendo o córtex e se estendendo para os tecidos moles circundantes. A extensão e as margens do fibroma são mais bem avaliadas com a ressonância magnética, sendo a técnica ideal para descrever o envolvimento dos tecidos moles. O fibroma desmoplásico geralmente mostra focos de intensidade de sinal baixo a intermediário em T2, que radiograficamente não corresponde à calcificação. 5 Além disso, apresenta realce heterogêneo após administração de contraste.

O estudo anatomopatológico forneceu o diagnóstico definitivo do tumor de nosso paciente. A OMS descreveu a aparência microscópica do fibroma desmoplásico como sendo composto de células fusiformes em uma matriz ricamente colagenosa. 1 O principal diagnóstico diferencial é um fibrossarcoma de baixo grau bem diferenciado. No entanto, o fibroma desmoplásico apresenta mínima atipia citológica, pleomorfismo e mitoses. Macroscopicamente, o tumor é firme e apresenta superfície branco-creme com padrão variegado em espiral.

A recorrência após curetagem simples foi relatada como muito alta; consequentemente, a excisão cirúrgica ampla é o tratamento atual de escolha. 6 7 8 9 Evans et al. 10 relataram uma taxa de recorrência após a ressecção intralesional de 33%, enquanto as técnicas mais agressivas mostraram uma taxa de 0%. Eles também afirmaram que a recorrência local foi fortemente associada à extensão dos tecidos moles. Böhm et al. 11 analisaram 189 pacientes e encontraram uma taxa de recorrência de 55-72% após curetagem simples e 0% após ressecção ampla. Além disso, destacaram o caráter agressivo que pode apresentar esta entidade devido ao seu risco de fratura patológica (12%) e alta taxa de recorrência.

Por outro lado, bons resultados após ressecção intralesional com menor morbidade pós-operatória também tem sido defendidos. 12 13 Alguns autores afirmaram que a adição de radioterapia adjuvante à curetagem intralesional pode ser uma abordagem alternativa quando a ressecção ampla levaria a um comprometimento funcional significativo. 14 Durante nossa abordagem cirúrgica agressiva, o paciente sofreu uma paralisia do nervo fibular comum e uma lesão vascular poplítea. A ressecção ampla em tumores maciços não é isenta de complicações. No entanto, preferimos essa abordagem porque o tamanho do tumor, a destruição óssea grave, a extensão dos tecidos moles e o envolvimento articular tornaram extremamente difícil realizar uma curetagem intralesional bem-sucedida sem comprometimento funcional ou recorrência local.

Relatamos o caso de um paciente de 45 anos com fibroma desmoplásico volumoso na tíbia proximal. Foi realizado com sucesso um procedimento cirúrgico em dois tempos: ressecção ampla e reconstrução endoprotética. Embora o fibroma desmoplásico seja um tumor benigno, os cirurgiões devem estar cientes da complexidade de seu tratamento nos casos localmente avançados e agressivos.
